# Radiation damage in protein crystallography at X-ray free-electron lasers

**DOI:** 10.1107/S2059798319000317

**Published:** 2019-01-28

**Authors:** Karol Nass

**Affiliations:** a Swiss Light Source, Paul Scherrer Institut, Forschungsstrasse 111, 5232 Villigen, Switzerland

**Keywords:** radiation damage, X-ray free-electron lasers, serial femtosecond crystallography

## Abstract

Radiation damage is still the most limiting factor in obtaining high-resolution structures of macromolecules in crystallographic experiments at synchrotrons. With the advent of X-ray free-electron lasers (XFELs) that produce ultrashort and highly intense X-ray pulses, it became possible to outrun most of the radiation-damage processes occurring in the sample during exposure to XFEL radiation. Although this is generally the case, several experimental and theoretical studies have indicated that structures from XFELs may not always be radiation-damage free. This is especially true when higher intensity pulses are used and protein molecules that contain heavy elements in their structures are studied. Here, the radiation-damage mechanisms that occur in samples exposed to XFEL pulses are summarized, results that show indications of radiation damage are reviewed and methods that can partially overcome it are discussed.

## Abstract 2

Research in the area of protein crystallography at X-ray free-electron laser sources is summarized from the perspective of radiation damage, including its mechanisms and effects, ways to minimize it and a comparison with the damage observable at synchrotrons.

## 1. Introduction   

Macromolecular X-ray crystallography (MX) has been the most powerful approach for obtaining three-dimensional structural information on biological species such as proteins, nucleic acids or viruses at up to atomic resolution which, together with functional studies, is crucial for understanding the mechanism underlying the given biological process (Shi, 2014 ▸). MX requires the use of radiation with wavelengths similar to or shorter than the length scale of atoms in order to yield high-resolution structures. However, this electromagnetic radiation causes damage to the sample as it carries sufficient energy to overcome the binding energies of electrons in atoms and molecules, which results in the ionization and excitation of the atoms in the specimen (Als-Nielsen & McMorrow, 2011 ▸).

The ratio of the number of elastically scattered to absorbed X-rays depends on the probabilities (cross-sections) of these interactions for specific atoms. The cross-section values for neutral atoms depend on the photon energy and the atomic number. From the cross-section values, for every scattered X-ray photon that contributes to the diffraction pattern many more are absorbed in the sample. The energy deposited in the sample owing to interactions of photons with electrons bound to atoms leads to the development of radiation-induced damage as the absorbed energy accumulates (Henderson, 1995 ▸). The primary causes of radiation damage are photo-absorption and inelastic interactions of X-rays with atoms (Henderson, 1995 ▸; Howells *et al.*, 2009 ▸). Electron-impact ionization cascades initiated by released and highly energetic photoelectrons and Auger electrons are created after initial photoabsorption and contribute to the evolution of damage in the sample (Ziaja *et al.*, 2002 ▸). They thermalize after a series of collisions with other electrons, and very reactive radical species are created that lead to a series of chemical reactions which further modify the protein crystal under investigation (O’Neill *et al.*, 2002 ▸; Wherland & Pecht, 2018 ▸). These changes accumulate as the energy deposited in the sample increases and typically cause a gradual decrease in the attainable resolution in the diffraction experiment, together with an increase in the Wilson *B* (temperature) factor, changes in the unit-cell parameters, the reduction of metals in the active sites and the elongation or breakage of disulfide bonds, as well as the modification of other specific sites of radiation-sensitive amino acids (Holton, 2009 ▸). Importantly, keeping the sample at a cryogenic temperature of around 100 K increases the tolerable dose roughly 70 times when compared with room temperature (Nave & Garman, 2005 ▸). Yet, redox-sensitive cofactors that contain heavier elements are particularly sensitive to damage owing to their increased likelihood of interaction with X-rays and secondary electrons created elsewhere in the sample. Additionally, heavy elements are characterized by an increased electrophilic nature, leading to the attraction of lower-energy electrons and subsequent reduction, compared with lighter elements (Beitlich *et al.*, 2007 ▸). For these reasons, radiation damage to biological samples initiated by X-rays is one of the main limiting factors in obtaining interpretable structural information from small and/or radiation-sensitive protein crystals. A comprehensive review of radiation damage in macromolecular crystallo­graphy at synchrotrons is given by Garman & Weik (2017 ▸).

X-ray free-electron lasers (XFELs) are novel X-ray sources that produce coherent and ultrashort (femtoseconds) pulses of light in the X-ray energy regime. The peak brightness of XFEL sources exceeds those of conventional laboratory and synchrotron X-ray sources that are suitable for MX applications by orders of magnitude (Patterson, 2014 ▸). It has been predicted (Neutze *et al.*, 2000 ▸) and verified experimentally (Chapman *et al.*, 2011 ▸) that diffraction data can be obtained using these highly intense and femtosecond duration pulses before signs of radiation damage are observed, at high resolution (Boutet *et al.*, 2012 ▸) and even when the dose absorbed by the crystal is orders of magnitude higher than the radiation-dose limits established as being safe at conventional X-ray sources (Garman & Weik, 2017 ▸). As the crystal is ultimately destroyed by the XFEL pulse after exposure, fresh sample or a part thereof needs to be constantly replenished for the next X-ray pulse and this serial approach to data collection is termed serial femtosecond crystallography (SFX; Schlichting, 2015 ▸). In terms of radiation damage, the main advantage of the currently available XFELs over synchrotrons is the increased tolerable dose limit owing to the fact that XFEL pulses terminate before the onset of disintegration of the crystal structure or before specific changes in atomic coordinates occur to an observable extent at a given resolution and photon flux density (Chapman *et al.*, 2014 ▸; Caleman *et al.*, 2015 ▸). Additionally, the creation, diffusion of and damage caused by radicals observed at other X-ray sources that require longer exposures is avoided on the timescales of XFEL pulses. Thus, XFELs enable the limitations imposed by radiation damage at traditional X-ray sources to be overcome and allow the determination of essentially undamaged structures of highly sensitive metalloproteins (Alonso-Mori *et al.*, 2012 ▸; Kern *et al.*, 2013 ▸; Hirata *et al.*, 2014 ▸; Young *et al.*, 2016 ▸; Suga *et al.*, 2017 ▸) and the structures of micrometre- to sub-micrometre-sized crystals (Redecke *et al.*, 2013 ▸; Colletier *et al.*, 2016 ▸; Gati *et al.*, 2017 ▸), including those of membrane proteins (Liu *et al.*, 2013 ▸). However, by using special experimental conditions such as a very high flux density arising from a nanometre-sized focus, long pulse durations or exotic modes of FEL operation that are not commonly used in SFX experiments, several studies have demonstrated that indications of damage caused by XFEL pulses can be observed in SFX data (Lomb *et al.*, 2011 ▸; Barty *et al.*, 2012 ▸; Nass *et al.*, 2015 ▸; Inoue *et al.*, 2016 ▸).

This work reviews the current knowledge on the mechanisms of ultrafast radiation damage to atoms in samples exposed to high-intensity XFEL radiation, compares the effects of radiation damage caused by XFEL pulses in protein crystals with the well known types of damage observed in MX at synchrotron sources, highlights studies that have observed indications of radiation damage in SFX data and discusses methods to minimize radiation damage at XFELs.

## 2. Mechanisms of radiation damage   

Photoabsorption is the main initiator of radiation damage in MX. It leads to the ionization of atoms and, in the case of XFELs, the high pulse intensity and ultrashort pulse duration cause excessive ionization of atoms in the sample that develops with time. As predicted by simulations, very high charge states of ions can be reached in the sample at the end of the XFEL pulse, which ultimately destroys the sample (Caleman, Bergh *et al.*, 2011 ▸; Hau-Riege, 2013 ▸). The X-ray pulse duration from an XFEL source is typically of the order of a few femto­seconds to a few tens of femtoseconds. Each pulse can contain up to 10^12^ photons. When a typical ∼30 fs pulse with 10^12^ photons of 12.4 keV energy (1 Å wavelength) is focused to an area of 1 µm^2^, the resulting surface power density at the sample is ∼6.6 × 10^18^ W cm^−2^, which corresponds to a photon density of approximately 10^4^ photons Å^−2^. Under such extreme irradiation, the consequence of exposure to a non-attenuated and tightly focused XFEL pulse is the rapid ionization of the atoms in the sample to high charge states via (multiple) photoabsorption(s) and electron-impact ionization (Caleman, Bergh *et al.*, 2011 ▸; Young *et al.*, 2010 ▸; Caleman, Huldt *et al.*, 2011 ▸). The ionization rate and the final level of ionization at the end of the pulse depend on the specific cross-sections, which depend on the atomic number, the photon energy and the current ionization state of an atom. The rapidly increasing ionization of atoms within the duration of an XFEL pulse will ultimately lead to destruction of the sample by Coulomb explosion. Therefore, the timescales of radiation damage in macromolecular crystallography at XFELs and synchrotrons are very different owing to differences in the exposure time and the photon flux density. Consequently, damage caused by chemically reactive species, which is one of the main sources of damage in MX at synchrotrons, is avoided at XFELs as the XFEL pulse terminates before these molecules are created; thus, the reactions that they cause in contact with other atoms are not present (Chapman *et al.*, 2014 ▸).

**Figure 1:**
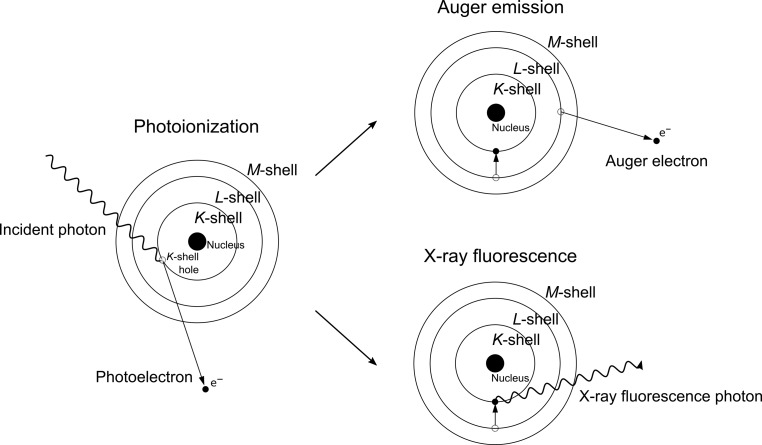
Illustration of the primary radiation-damage mechanisms that occur in samples exposed to X-rays. Photoionization typically initiates the damage by removing an inner-shell electron from an atom. Such a photoionized atom exists in an excited state that can relax via one of two pathways: Auger emission or X-ray fluorescence. The first is more probable for lighter elements, whereas the latter is more probable for heavier elements.

On the timescale of the XFEL pulse duration, radiation damage mostly occurs owing to photoabsorption and electron-impact ionization. The energy of an X-ray photon is absorbed by an atom and an inner-shell electron (photoelectron) is ejected, which carries away the photon energy reduced by the binding energy of the electron. Such an ionized atom now exists in an excited state that can decay via one of two main pathways (Fig. 1 ▸). The first relaxation pathway is the Auger relaxation (decay) process, during which the core-hole that was created after the inner-shell electron was ejected is refilled by an electron from the outer shell. The energy difference between the outer- and inner-shell electron levels involved in the Auger process is then carried away by the removal of another electron from an outer shell (Auger electron), leaving the atom doubly ionized (Als-Nielsen & McMorrow, 2011 ▸). The time that it takes to complete this process varies with the atom type and ionization level: the typical timescale of the Auger relaxation process is between 0.1 and 10 fs (Campbell & Papp, 2001 ▸). The Auger process takes longer in lighter elements than in heavier elements, and it can be prolonged if the atom is already ionized when another photoabsorption occurs (Young *et al.*, 2010 ▸). Auger decay is a more favourable relaxation process for lighter elements than for heavier elements. In heavier elements the second possible relaxation process, emission of an X-ray fluorescence photon, is the more probable pathway to dissipate the energy of an excited state of an atom that remains after photoabsorption. In this process, the core-hole that is left after photoabsorption is filled by an outer-shell electron, and a fluorescence photon with an energy equal to the difference between the two involved electronic orbitals is released, leaving the atom only singly ionized, compared with the Auger relaxation process, which leaves it doubly ionized (see above). However, the outer-shell hole that is left after the X-ray fluorescence photon is released decays further through a cascade of Auger processes. Therefore, photoionization leads to the ejection of more electrons per atom in heavier elements than in lighter elements (Santra & Young, 2014 ▸).

**Figure 2:**
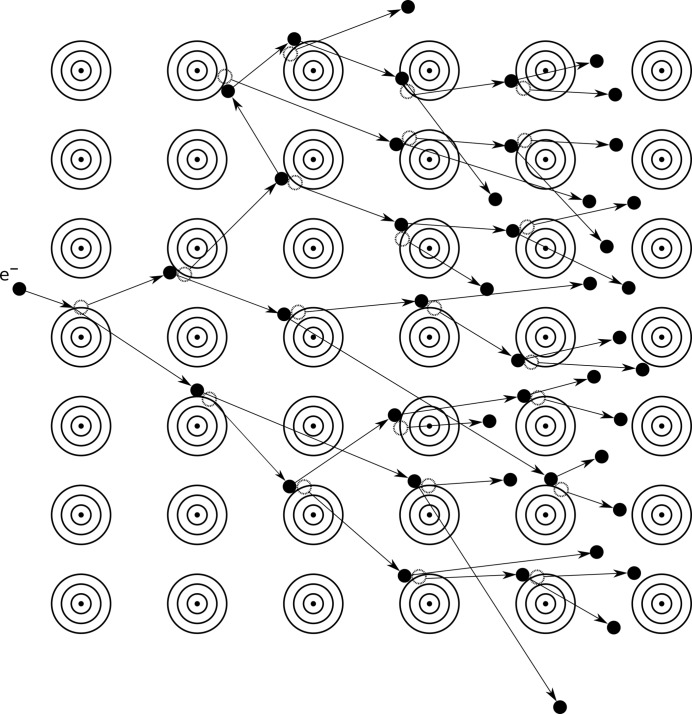
Illustration of the secondary radiation-damage mechanism induced by intense XFEL radiation in protein crystals. The electron-impact ionization cascades are initiated by released photoelectrons or Auger electrons and significantly contribute to the increase of the ionization level and the temperature in the sample. Electron-impact ionization cascades can add several hundreds of ionizations to the primary X-ray-induced damage, and can reach a radius of several hundreds of nanometres in a few tens of femtoseconds before thermalization.

Released photoelectrons and Auger electrons have sufficient energy to ionize surrounding atoms by direct collisions (Ziaja *et al.*, 2002 ▸). The energy of a photoelectron ejected from the *K* shell is equal to the X-ray photon energy minus the binding energy of the *K*-shell electron. For carbon, nitrogen and oxygen this energy is of the order of 300–600 eV (Cardona & Ley, 1978 ▸). This means that the photoelectron will have an energy almost equal to the energy of the incoming X-ray, so it will travel rapidly through the sample, colliding with and removing many electrons from other atoms until it thermalizes. The energy of a released Auger electron, on the other hand, is more than an order of magnitude smaller, at approximately 250–500 eV (Bruch *et al.*, 1985 ▸). Nevertheless, it will also collide with bound electrons and remove them from atoms, requiring proportionally fewer collisions than photoelectrons to thermalize. Consequently, the released photo and Auger electrons initiate photoelectron- and Auger electron-impact ionization cascades as they collide with other electrons, which rapidly increases the ionization level of atoms in the sample (Fig. 2 ▸). For example, the impact ionization cascades from photoelectrons created by 8 keV photons can fully develop in less than 30 fs, reaching a radius of ∼500 nm (Caleman, Huldt *et al.*, 2011 ▸). Cascades from Auger electrons that originate from C, N or O atoms thermalize faster, in approximately 3–5 fs, and only reach a radius of gyration of ∼10 nm (Caleman, Huldt *et al.*, 2011 ▸). The number of ionizations in the complete Auger cascade is ∼20 and that in the photoelectron cascade is ∼400 (Caleman, Huldt *et al.*, 2011 ▸; Caleman *et al.*, 2009 ▸; Cowan & Nave, 2008 ▸; Nave & Hill, 2005 ▸; Hau-Riege *et al.*, 2004 ▸; O’Neill *et al.*, 2002 ▸). When an XFEL pulse is focused to an area of 1 µm^2^ on a typical protein crystal with an average organic atomic composition, the absorbed dose can reach up to 2.3 GGy, which can heat it to more than 400 000 K during the duration of the pulse (Chapman *et al.*, 2014 ▸). The large number of ionizations that develop during the pulse duration in the sample can lead to the formation of hot plasma (Chapman *et al.*, 2014 ▸). Interestingly, the interaction between the very high electric field strengths (∼6 × 10^5^ V Å^−1^) resulting from an extreme number of photons per atom (>10^9^ photons Å^−2^) and atoms in the sample was predicted to cause plasma formation in less than 1 fs owing to an ‘inverse bremsstrahlung’ effect (Doniach, 1996 ▸).

## 3. Global radiation damage at XFELs   

When the ionization level in the crystal is high enough for the ions to feel each other’s electrostatic potential, they start moving away from each other owing to repulsive forces (Barty *et al.*, 2012 ▸). This movement initiates a gradual increase of the disorder parameter of the crystal structure during the pulse duration, which results in a decay of Bragg peak intensities at high scattering angles. Consequently, Bragg peaks at low scattering angles will be observed for longer and thus accumulate more scattered photons than those at high angles. The outcome of this effect is similar to that of the global damage effects observed in macromolecular crystallography at synchrotrons, where the high-resolution diffraction spots disappear from consecutive diffraction images as the dose absorbed by the crystals increases. The difference is that the temporal evolution of this damage effect is recorded in a single frame at an XFEL, whereas it can be spread over multiple frames at a synchrotron. It has been predicted that if the damage was distributed uniformly within the asymmetric units of the crystals, then it could be possible to correct for it by scaling (Barty *et al.*, 2012 ▸). However, another study found that such scaling of data is difficult to accomplish, and attributed this complexity to possible non-uniformity of the radiation-damage dynamics across the sample and argued for the existence of ‘hot spots’ of damage (Lomb *et al.*, 2011 ▸). In addition to induced disorder, changes in the atomic scattering form factors during the pulse can occur owing to the complex ionization dynamics of different atom types during the pulse, which can modify the scattered intensities (Hau-Riege, 2007 ▸). For example, at one ionization per atom on average, the scattering power of atoms is reduced, leading to a decrease of up to 30% in the scattered intensity (Caleman, Huldt *et al.*, 2011 ▸).

A study aimed at observing global radiation damage to the crystalline structure of thin diamond films was performed at SACLA (Ishikawa *et al.*, 2012 ▸). A special two-colour double-pulse operation mode of the SACLA XFEL (Ishikawa *et al.*, 2012 ▸; Tono *et al.*, 2013 ▸) was used in an X-ray pump/X-ray probe diffraction experiment using a nanocrystalline diamond film as a target for observing temporal evolution of structural damage in a crystalline structure (Inoue *et al.*, 2016 ▸). Two X-ray pulses of 6.1 and 5.9 keV were generated by tuning the undulator gaps of upstream and downstream parts of the undulator, and the time between the pulses was controlled by a magnetic chicane between the undulator parts with an accuracy of 0.1 fs. The pulses were spatially overlapping and were focused to ∼130 × 200 nm, which resulted in intensity of ∼10^19^ W cm^−2^ per pulse. The time delays between the X-ray pump and X-ray probe pulses ranged from 0.3 to 80 fs. This setup allowed the recorded intensities of two spatially separated Bragg reflections from diamond to be analysed as a function of the time delay between the pulses. The analysis revealed a decrease in the intensity of the 111 and 220 reflections after a time delay of 20 fs. This was attributed to structural damage and not to electronic damage because of the low ionization level under these experimental conditions. Interestingly, after the initiation of atomic movement, the rates of the estimated atomic displacements of C atoms perpendicular to the two crystallographic planes were different, which could not be explained just by the increase in the global Debye–Waller factor. This indicated that complex dynamics of structural damage may take place during irradiation by intense XFEL pulses even in homogenous materials such as diamond.

## 4. Specific radiation damage at XFELs   

**Figure 3:**
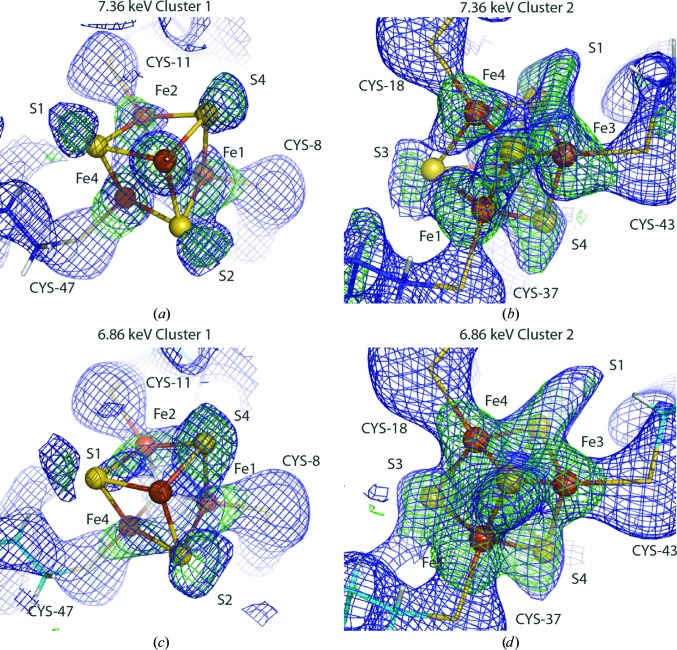
The two [4Fe–4S] clusters (ball-and-stick representation) in ferredoxin; the 2*mF*_obs_ − *DF*_calc_ (blue, 1.0σ) and *F*_obs_ − *DF*_calc_ (green, 2.5σ) electron-density maps show indications of reproducible, localized radiation damage to heavy-atom centres in protein crystals exposed to intense XFEL radiation. The two clusters show different levels of damage, indicating that the local environment may play a role in radiation-damage dynamics at XFELs. The effects at photon energies of 7.36 and 6.86 keV (above and below the Fe *K* absorption edge) on the reconstructed electron-density maps of the [4Fe–4S] clusters are similar. Reproduced from Nass *et al.* (2015 ▸).

It has been estimated that to ionize every atom in a protein crystal of an average composition at the end of a typical XFEL pulse once, an absorbed dose of 400 MGy is required (Chapman *et al.*, 2014 ▸). Since scattering and ionization occur during the entire pulse duration, it is assumed that the scattering signal is obtained from atoms mostly in their intact (pristine) state and that this dose can be used as a threshold marker to obtain signal before any modification of the electronic structure of the sample has occurred (Kern *et al.*, 2015 ▸). Nevertheless, on timescales of several to tens of femtoseconds photoionization of atoms cannot be avoided. Elements with higher atomic numbers such as iron or sulfur are more susceptible to X-ray-induced damage by photoelectron- and electron-impact ionization because they are characterized by higher atomic cross-sections for this type of interaction than lighter elements such as carbon, nitrogen and oxygen, which are the main components of proteins (Henke *et al.*, 1993 ▸). Therefore, it has been predicted that heavy atoms and atoms in their vicinity could form areas of increased localized structural and electronic damage compared with the rest of the protein (Jurek & Faigel, 2009 ▸; Hau-Riege *et al.*, 2004 ▸) and create effects of charge migration from lighter elements to rapidly ionizing heavier elements (Erk *et al.*, 2013 ▸, 2014 ▸). Consequently, these regions are more likely to be structurally damaged than the rest of the protein. Importantly, metalloproteins containing a metal cofactor are involved in many essential processes (*e.g.* photosynthesis and respiration). Indications of such localized structural and electronic damage have been obtained experimentally (Nass *et al.*, 2015 ▸) and have been predicted by simulations (Hau-Riege & Bennion, 2015 ▸) when using specific conditions. Pulses with a photon energy above the absorption edge of the metal atom in the active site, iron in this case, were focused to submicrometre dimensions and the pulse duration was slightly longer (80 fs) than that typically used in SFX experiments. The ionized S atoms of the 4Fe–4S iron–sulfur clusters in ferredoxin crystals moved in specific directions owing to repulsion forces, as favoured by their geo­metrical arrangement (Hau-Riege & Bennion, 2015 ▸; Nass *et al.*, 2015 ▸). In the experimental results, one of the two 4Fe–4S clusters in the structure appeared to be more damaged than the other, indicating that the local protein environment plays a role in damage dynamics (Fig. 3 ▸). The increased sensitivity to damage selectivity of heavy elements in protein crystal structures has been used to propose a new phasing method, in which high-intensity XFEL pulses selectively modify the electronic structure of heavy atoms in the protein. This results in shifts of element-specific X-ray absorption edges; therefore, the peak and remote data sets typical for a MAD experiment could be recorded at the same wavelength with high and low pulse intensity (Son, Chapman *et al.*, 2011 ▸). Recently, a theoretical study focused on exploring the possibility of mapping the non-uniform ion distribution in a protein as it undergoes the Coulomb explosion following intense ionization for applications in orientation recovery in single-particle imaging experiments at XFELs was published (Östlin *et al.*, 2018 ▸). It showed the existence of localized hot and cold ‘spots’ of ion density in a protein exposed to an XFEL pulse of high intensity and that the predicted reproducibility of trajectories of carbon and sulfur ions in lysozyme exposed to XFEL radiation varied.

## 5. Requirements for outrunning radiation damage   

It may be possible to reduce the number of multiple photoabsorption events per atom and consequently the level of ionization in the sample by using pulses shorter than the lifetime of the Auger decay processes. After the initial photoionization and Auger relaxation is complete, two electrons are removed from the outer shells of an atom, leading to a doubly charged ion with all inner-shell electrons filled that is ready for the next photoionization event. However, when the pulses are shorter than the Auger decay lifetime, the generation of high-charge ions is suppressed. For lighter elements, the relatively slow Auger decay process is more favourable than the faster fluorescence relaxation pathway; therefore, when using pulses shorter than Auger processes the ionization level of lighter elements at the end of the pulse can be reduced. This phenomenon has been called frustrated X-ray absorption or intensity-induced X-ray transparency (Young *et al.*, 2010 ▸; Hoener *et al.*, 2010 ▸). In this phenomenon the production of high charge states of atoms via multiple photo­absorptions is suppressed in comparison to longer pulse durations because the core-hole that is left after the first absorption event is not filled for as long as the Auger decay lifetime lasts, reducing the number of inner-shell electrons available for X-ray absorption. For example, the measured lifetime of the Auger decay for iron is 0.55 fs, that for sulfur is 1.3 fs and that for carbon is 10 fs (Campbell & Papp, 2001 ▸). In contrast, when the XFEL pulse is longer than the Auger lifetime, core-excited states have sufficient time to decay, which results in the refilling of inner-shell holes with electrons from outer shells, and sequential multi-photon ionization can occur, possibly removing all electrons from an atom if the pulse is sufficiently intense and long (Young *et al.*, 2010 ▸). Using X-ray pulses shorter than the Auger lifetime of atoms has another advantage for reducing radiation damage by outrunning the development of secondary electron-impact cascades. It has been estimated that one 6 keV photoelectron will lead to the creation of ∼300 secondary electrons via impact ionization cascades before the secondary electrons thermalize (Caleman, Huldt *et al.*, 2011 ▸). Most impact ionization cascades will be completed after tens of femtoseconds; therefore, electron-impact ionization cascades caused by highly energetic photoelectrons released after the initial X-ray absorption would not have enough time to fully develop if sufficiently short pulses were used. This would result in a reduction of the ionization level in the sample and in the reduction of the radiation damage observable during the X-ray pulse. In order to completely outrun the creation of electron-impact ionization cascades created by photoelectrons, the pulse needs to be shorter than the time it takes for the first collision to occur, which depends on the energy of the photoelectron and is typically much less than 1 fs (Son, Young *et al.*, 2011 ▸).

## 6. Conclusions and outlook   

In this review, an overview of radiation-damage processes occurring on ultrafast timescales and a summary of published research articles that have investigated the radiation-damage processes occurring in samples exposed to high-intensity XFEL pulses have been presented. In contrast to decades of research in the field of radiation damage in macromolecular crystallo­graphy at conventional X-ray sources, only a handful of articles have investigated this phenomenon at X-ray free-electron lasers. It appears that in the case of SFX most studies have not observed damage effects in electron-density maps or in the X-ray emission spectra when using modest photon flux densities. The degree to which radiation damage will modify protein structures obtained from experiments that use higher flux densities (>10^19^ W cm^−2^) with pulses focused to sub­micrometre dimensions and shorter pulse durations on the few femtoseconds and subfemtosecond timescales remains to be explored. As the number of available XFEL sources and the user community grows, it is expected that this research field will also advance, allowing us to better understand the nature of radiation damage at XFELs and to aid the development of methods to overcome it.
